# Combating Genetic Heterogeneity for Polygenic Prediction of Susceptibility to Brain β-Amyloid Deposition: Beyond *APOE*

**DOI:** 10.1212/NXG.0000000000200266

**Published:** 2025-07-21

**Authors:** Vijay K. Ramanan, Michael G. Heckman, Ekaterina I. Hofrenning, Scott A. Przybelski, Jonathan Graff-Radford, Val J. Lowe, Mary M. Machulda, Melissa E. Murray, Alicia Algeciras-Schimnich, Daniel J. Figdore, David A. Bennett, David S. Knopman, Clifford R. Jack, Ronald C. Petersen, Owen A. Ross, Prashanthi Vemuri, for the Alzheimer's Disease Neuroimaging Initiative, Michael Weiner

**Affiliations:** 1 Department of Neurology, Mayo Clinic Rochester, Rochester, MN;; 2 Department of Quantitative Health Sciences, Mayo Clinic Jacksonville, Jacksonville, FL;; 3 Department of Quantitative Health Sciences, Mayo Clinic Rochester, Rochester, MN;; 4 Department of Radiology, Mayo Clinic Rochester, Rochester, MN;; 5 Department of Psychiatry and Psychology, Mayo Clinic Rochester, Rochester, MN;; 6 Department of Neuroscience, Mayo Clinic Jacksonville, Jacksonville, FL;; 7 Department of Laboratory Medicine and Pathology, Mayo Clinic Rochester, Rochester, MN;; 8 Rush Alzheimer's Disease Center, Rush University Medical Center, Chicago, IL; and; 9 Departments of Neuroscience and Clinical Genomics, Mayo Clinic Jacksonville, Jacksonville, FL.

## Abstract

**Background and Objectives:**

The *APOE* (apolipoprotein E) ε4 allele is the strongest known genetic risk factor for sporadic Alzheimer disease (AD) and for brain amyloidosis, an early marker of disease pathophysiology. However, *APOE* ε4 is present in only 25% of the general population and is by itself inadequate for explaining susceptibility to amyloid accumulation or AD diagnosis. Existing studies have been limited by potential confounding due to inclusion of individuals carrying *APOE* ε4 or ε2 (which has a modest protective association). We hypothesized that genome-wide association study (GWAS) and genetic risk score (GRS) analyses in *APOE* ε3/ε3 individuals would uniquely identify novel predictors of β-amyloid pathology in older adults.

**Methods:**

We analyzed data from the Mayo Clinic Study of Aging (MCSA), Alzheimer's Disease Neuroimaging Initiative (ADNI), and Rush Religious Orders Study and Memory and Aging Project. Frequency of *APOE* ε3/ε3 in those samples ranged from 48% to 61%. A GWAS was performed across 1,496 individuals with amyloid PET to identify candidate variants for GRS generation. Postmortem neuropathologic data (N = 710) were used to refine the variant list to capture high-likelihood true associations. An independent sample (N = 641) with plasma p-tau_181_ data was used for validation.

**Results:**

The GWAS identified previously implicated (e.g., *PICALM* and *RBFOX1*) and novel potential associations with amyloid PET burden. A non-*APOE* GRS of top variants was strongly associated with amyloid PET levels in the MCSA (*p* = 4.34 × 10^−9^, β = 5.88) and ADNI (*p* = 1.87 × 10^−8^, β = 12.1) cohorts. In both cohorts, this non-*APOE* amyloid GRS outperformed a comparator GRS (based on variants associated with clinically diagnosed AD dementia risk) in explaining phenotypic variation. The non-*APOE* amyloid GRS was also associated with postmortem neuropathologic β-amyloid and neurofibrillary tangle burden and in an independent sample was associated with plasma p-tau_181_ concentrations (a robust indicator of cerebral amyloidosis).

**Discussion:**

Our non-*APOE* amyloid GRS, which appropriately includes variants associated with amyloid deposition in *APOE* ɛ4/ɛ2 noncarriers, may advance personalized prediction of genetic susceptibility to β-amyloid accumulation within the large segment of the population that is *APOE* ε3/ε3. This may have future implications for risk modification, trial enrollment, and treatment selection.

## Introduction

The most common isoform of the apolipoprotein E (*APOE*) gene is the ε3 allele, which is considered neutral regarding risk of Alzheimer disease (AD).^1^ In the general population, the *APOE* ε4 allele is the strongest known genetic risk factor of sporadic AD, and its prevalence increases from 25% overall to 40%–65% in patients with AD dementia.^2^ *APOE* ε4 also has a robust association with brain amyloidosis, which is a key early pathophysiologic event in AD.^3^ However, *APOE* ε4 is neither necessary nor sufficient for the development of AD dementia, highlighting that additional factors influence susceptibility to disease.^4^ The *APOE* ε2 allele has a modest protective association against AD dementia risk, which is hypothesized to relate to amyloid-dependent and amyloid-independent mechanisms.^5^ However, *APOE* ε2 is also associated with increased risk of certain non-AD disorders and is itself not sufficient to account for protection against AD dementia.^5^ Given that *APOE* ε3/ε3 individuals account for most (50%–70%) of the worldwide population,^6^ there is a pressing need to develop risk stratification tools that extend beyond *APOE* to offer broader clinical utility.

Alongside *APOE*, case-control genome-wide association studies (GWASs) have identified additional risk variants for clinically diagnosed AD dementia.^7,8^ However, there is concern that the outsized influence of *APOE* variants on AD risk may mask true associations of novel genetic loci, particularly if individuals carrying *APOE* ε4 or ε2 are included in analyses.^9^ In response, case-control GWASs and sequencing-based studies stratified by *APOE* ε4 status have been pursued.^9-11^ Nevertheless, these approaches are limited by a lack of biomarker data, given that there is nontrivial discordance between biologically defined AD and clinically diagnosed AD dementia, which can affect genotype-phenotype associations. Extant literature supports the concept that the genetic architecture influencing risk of clinically diagnosed AD dementia appears to be quite distinct from the genetic factors underlying key AD endophenotypes.^3,12-19^

We hypothesized that a GWAS of brain β-amyloid burden in *APOE* ε3/ε3 individuals would uniquely capture genetic variation associated with an early pathophysiologic event in AD, which is now being targeted by new disease-modifying therapies.^20^ To these data, we applied a genetic risk score (GRS) framework to generate and validate an aggregate measure of genetic susceptibility to amyloidosis, relevant to the large proportion of the population not carrying the *APOE* ε4 or ε2 alleles.

## Methods

### Cohort/Sample Characteristics

The Mayo Clinic Study of Aging (MCSA) is a multimodal, population-based, prospective study of older adults in Olmsted County, Minnesota,^21^ with recruitment linked to the Rochester Epidemiology Project (REP) database.^22^ The Alzheimer's Disease Neuroimaging Initiative (ADNI) is a longitudinal multicenter study to facilitate development of biomarkers for the early detection and tracking of AD.^23,24^ The Rush Religious Orders Study (ROS) is a longitudinal study of older religious clergy without known dementia at enrollment.^25^ The Rush Memory and Aging Project (MAP) is a longitudinal study of older adults recruited from the Chicago metropolitan area.^26^ Data from the ROS and MAP samples were analyzed together (ROS/MAP) because of the harmonized protocols across these 2 studies centered at the same site.

### Standard Protocol Approvals, Registrations, and Patient Consents

All study protocols for the MCSA, ADNI, ROS, and MAP were approved by each participating site's institutional review boards. Written informed consent to participate in the research studies was obtained from all participants or their surrogates. ROS/MAP participants also signed an Anatomic Gift Act for organ donation and a repository consent allowing their data to be shared.

### Single-Nucleotide Variant (SNV) Array Data

In the main MCSA sample, SNV array data were acquired using the Illumina Infinium Global Screening Array-24 v2.0.^27^ ADNI participants were genotyped on one of 3 Illumina GWAS arrays,^28^ with postprocessed data files downloaded from the ADNI database. Genotype data for ROS/MAP participants were generated via the Affymetrix GeneChip 6.0,^29^ and postprocessed data files were obtained from the AD Knowledge Portal. For quality control, SNV-level exclusion criteria included call rate <95%, Hardy-Weinberg equilibrium *p* < 1 × 10^−5^, or minor allele frequency (MAF) < 1%. Sample-level exclusion criteria included overall call rate <98%, chromosomal call rate <50%, discordance of inferred sex with clinical data, or evidence of significant relatedness defined by PLINK identity-by-descent analysis (PI_HAT ≥0.25). Analyses were restricted to individuals with European ancestry to minimize spurious effects from population heterogeneity, and principal component eigenvectors were generated for use as covariates to account for any residual effects of population stratification. Following these steps, array genotype data were available for 1,727 MCSA, 1,661 ADNI, and 1,700 ROS/MAP participants. For validation, subsequent analyses were performed using an independent set of MCSA participants for whom genotype data were obtained separately, using a custom automated workflow for genotyping-by-sequencing (GxS) developed at Regeneron Corporation (Tarrytown, NY).^30^

### Genome-Wide Imputation

The TOPMed Imputation Server and TOPMed GRCh38/hg38 build reference panel^31^ were used for genome-wide imputation within the MCSA, ADNI (in 3 separate batches per GWAS array, followed by merging across the ADNI sample), and ROS/MAP (in one batch because of a single GWAS array backbone) cohorts. The imputation server used Minimac4, and Eagle v2.4 was used for phasing. Variants with low imputation quality (*r*^*2*^ < 0.8) were filtered out. Additional standard quality control filters were applied, with exclusion of samples having a call rate <98% and with exclusion of SNVs having a genotyping rate <95%, Hardy-Weinberg equilibrium *p* < 1 × 10^−6^, or monomorphic genotype. For this study, given the sample sizes of the cohorts, we focused on SNVs with MAF ≥5% to limit the potential for undue influence from uncommon variants.

### Neuroimaging Data

Cross-sectional global cortical β-amyloid PET load was measured on the Centiloid (CL) scale for standardization across data sets.^32^ Amyloid PET scans in the MCSA cohort were performed with ^11^C-Pittsburgh compound B (PiB) and were analyzed using an in-house fully automated image processing pipeline.^33^ In the ADNI cohort, amyloid PET was performed with ^18^F-florbetapir (AV-45) with acquisition and processing protocols described in detail at the ADNI website.

### Postmortem Neuropathologic Data

Postmortem β-amyloid burden was characterized in the ROS/MAP sample by the percent area of cortex occupied by β-amyloid protein (via immunohistochemistry and quantified by image analysis) across 8 brain regions, as described previously.^34^ Mean neuronal neurofibrillary tangle density across the same 8 brain regions was determined via molecularly specific immunohistochemistry using antibodies to hyperphosphorylated tau (AT8).^34^ In the MCSA cohort, standardized sampling and dissection protocols were used, with formalin-fixed and paraffin-embedded tissue sections cut at 5-μm thickness for histologic and immunohistochemical studies and with Braak neurofibrillary tangle stage assessed using either phospho-tau antibody (AT8; 1:1,000; Endogen, Woburn, MA) or modified Bielschowsky silver stain.^35^

### Plasma Biomarker Data

In the MCSA cohort, plasma tau_181_ was measured from stored blood samples using the Quanterix HD-X analyzer (Quanterix Corporation, Lexington, MA) and SiMoA Advantage V2 kit (item #103714). Blood sample collection and processing and assay calibration and quality control were completed as described previously.^36^ For plasma p-tau_181_, the percentage coefficient of variation (measure of interassay imprecision) was 6% at an approximate concentration of 3.7 pg/mL and 5% at an approximate concentration of 119 pg/mL.

### Statistical Analyses

**Figure 1:**
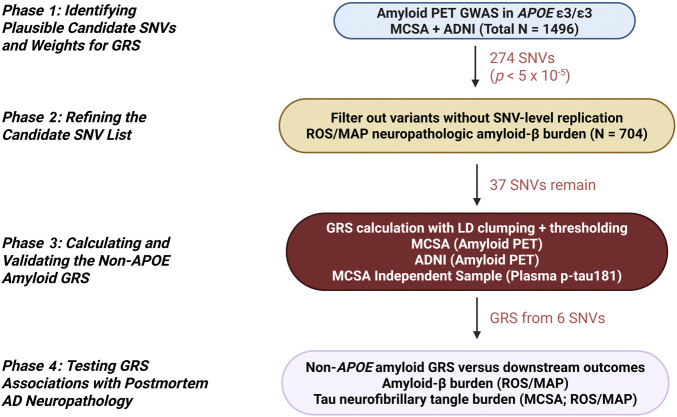
Summary of Study Design A flowchart describes the overall study design and the analytical phases for generating and validating the non-*APOE* amyloid genetic risk score (GRS). Created in BioRender. MD, V. (2025) BioRender.com/qri2rns.

The overall study design is illustrated in Figure 1. The overarching goal of the analyses was to generate a non-*APOE* GRS for β-amyloid pathology through multicohort analyses of *APOE* ε3/ε3 individuals. For the primary outcome measures analyzed, *APOE* ε3/ε3 individuals comprised 54% (983/1,696) of the possible MCSA GWAS–amyloid PET sample, 48% (513/1,068) of the possible ADNI GWAS–amyloid PET sample, 61% (710/1,158) of the possible ROS/MAP GWAS–neuropathology sample, and 58% (641/1,105) of the possible MCSA GWAS–plasma biomarker validation sample.

#### Phase 1: Identifying Candidate SNVs

PLINK (version 1.9)^37^ was used to perform a GWAS of amyloid PET burden in the MCSA cohort (N = 983) and separately in the ADNI cohort (N = 513). Random-effects inverse variance weighted meta-analysis was then performed across the 2 cohorts (total N = 1,496). For these analyses, linear regression with an additive genetic model was used and age at scan, sex, and the first 5 genetic principal components were included as covariates. This phase yielded a set of SNVs displaying suggestive association with amyloid PET burden (*p* < 5 × 10^−5^) to be used as candidates for entry into the GRS.

#### Phase 2: Refining the Candidate SNV List

To enhance the likelihood that variants included in the GRS reflected true associations with β-amyloid accumulation, we refined the candidate SNV list using data from an independent sample (ROS/MAP) with postmortem neuropathologic assessment of β-amyloid burden (sample N = 710). Specifically, linear regression was performed using the composite measure of β-amyloid burden (described above) as the outcome and including age at death, sex, and the first 5 genetic principal components as covariates. The candidate SNV list was then filtered to exclude those SNVs displaying *p* ≥ 0.2 in the neuropathology sample or a discordant direction of effect across the amyloid PET meta-analysis and the ROS/MAP neuropathologic analysis.

#### Phase 3: Calculating and Validating the Non-*APOE* Amyloid GRS

Applying the SNV list from Phase 2, PRSice-2^38^ was used to calculate a GRS associated with amyloid PET burden in the MCSA and ADNI samples. The PRSice-2 algorithm uses a clumping and thresholding (“C + T”) method, where the input SNVs are pruned to account for linkage disequilibrium (LD; retaining only the top associated variant for any pairs with *r*^*2*^ ≥ 0.1) and a threshold for SNV entry (prespecified at *p* < 5 × 10^−5^ as noted above). Age at scan, sex, and the first 5 genetic principal components were included as covariates, and the weight for each SNV in the model was based on its univariate linear regression coefficient in the initial amyloid PET meta-analysis. For benchmarking, we calculated an AD GRS based on the variants (excluding the *APOE* region) identified in a recent large case-control GWAS as associated with risk of clinically probable AD dementia.^7^ We then compared the performance of this AD GRS with the novel non-*APOE* amyloid GRS generated through this work.

For independent validation, we also tested the non-*APOE* amyloid GRS for association with plasma p-tau_181_ concentrations (as a blood-based biomarker of cerebral amyloidosis)^39^ in 641 *APOE* ε3/ε3 MCSA participants who were not included in the initial amyloid PET data set used for discovery. For the plasma biomarker replication analyses, individuals who were identified through medical record abstraction to have chronic kidney disease (CKD) were excluded to avoid potential confounding, given the relationship between CKD and plasma p-tau_181_ concentrations.^40^

#### Phase 4: Testing the Non-*APOE* Amyloid GRS for Associations With Postmortem AD Neuropathology

In ROS/MAP participants, the non-*APOE* GRS was tested for association with postmortem β-amyloid burden and neurofibrillary tangle density using linear regression, including age at death, sex, and the first 5 genetic principal components as covariates. In MCSA participants, logistic regression was used to test for association of the non-*APOE* GRS with high postmortem neurofibrillary tangle burden (defined by Braak stage ≥4), including age at death, sex, and the first 5 genetic principal components as covariates.

### Data Availability

Deidentified data from this study are available from the authors on reasonable request.

## Results

### Sample Characteristics

The samples analyzed from each cohort were distinct in certain characteristics (Table 1 and eTable 1). Most MCSA participants were cognitively unimpaired at the time of amyloid PET while most ADNI participants had diagnoses of mild cognitive impairment (MCI) or dementia at the time of amyloid PET. Most of the MCSA and ADNI participants were men while women represented the preponderance of the ROS/MAP sample. There were differences in age across the cohorts (*p* < 0.001), with ROS/MAP participants being the oldest and MCSA participants being the youngest. On average, ADNI and ROS/MAP participants had more years of education than MCSA participants (*p* < 0.001).

**Table 1:** Sample Characteristics

	MCSA (N = 983)	ADNI (N = 513)	ROS/MAP (N = 710)	MCSA replication (N = 641)
Age (y)	73.3 (10.3)	75.0 (7.6)	^a^80.2(6.9)	71.2 (17.5)
Sex	M - 529 (54%) W - 454 (46%)	M - 273 (53%) W - 240 (47%)	M - 237 (33%) W - 473 (67%)	M - 317 (50%) W - 324 (50%)
Education (y)	14.7 (2.7)	16.4 (2.6)	16.3 (3.7)	14.5 (2.6)
Diagnosis	^b^857 (88%) CU 102 (10%) MCI 14 (1%) DEM	210 (41%) CU 243 (47%) MCI 60 (12%) DEM	^c^238 (34%) CU 184 (26%) MCI 288 (41%) DEM	^d^381 (59%) CU 238 (37%) MCI 21 (3%) DEM
Age at death (y)	^d^89.6 (6.2)	—	^e^89.9 (6.4)	—
Amyloid status via PET	679 (69%) NEG 304 (31%) POS	323 (63%) NEG 190 (37%) POS	—	—
Amyloid PET centiloids	36.6 (32.6)	27.0 (40.9)	—	
Plasma p-tau181 (pg/mL)				2.34 (1.61)

Abbreviations: ADNI = Alzheimer's Disease Neuroimaging Initiative; CU = cognitively unimpaired; DEM = dementia; M = men; MCI = mild cognitive impairment; MCSA = Mayo Clinic Study of Aging; NEG = negative; POS = positive (abnormal); W = women.

Values displayed as mean (SD) or number (percentage).

a Age at baseline visit.

b Missing for 10 individuals in the MCSA sample.

c Consensus diagnosis at time of death (if available).

d Unspecified for 1 individual in the MCSA replication sample.

e For those included in postmortem neuropathologic analyses (MCSA: N = 63; ROS/MAP: N = 704).

#### Phase 1: Identifying Candidate SNVs—GWAS of Amyloid PET

**Figure 2:**
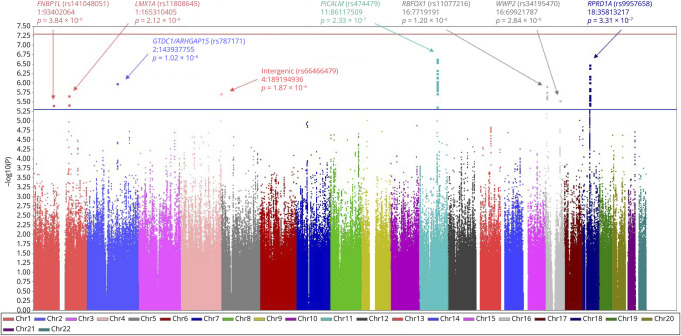
Manhattan Plot of GWAS Meta-Analysis of Amyloid PET in *APOE* ε3/ε3 Participants Observed -log_10_ *p* values are displayed (y-axis) for all SNVs tested in the GWAS meta-analysis of amyloid PET burden across *APOE* ε3/ε3 participants from the MCSA and ADNI cohorts (total N = 1,496). No genome-wide significant associations (*p* < 5 × 10^−8^) were identified (red line). A total of 8 independent loci displayed suggestive association *p* < 5 × 10^−6^ (blue line) in the meta-analysis, with top findings annotated in the figure. ADNI = Alzheimer's Disease Neuroimaging Initiative; GWAS = genome-wide association study; MCSA = Mayo Clinic Study of Aging.

GWAS of amyloid PET burden revealed no genome-wide significant associations in the ADNI or MCSA discovery cohorts. There was no evidence of systematic inflation of *p* values in the analysis (λ_ADNI_ = 1.007, λ_MCSA_ = 1.000) samples. Meta-analysis across the 2 cohorts (total N = 1,496) identified 8 independent loci with association *p* < 5 × 10^−6^ (Figure 2). The top association was for rs474479 (*p* = 2.33 × 10^−7^, β = −6.40), which is proximal to and represents a known expression quantitative trait locus (eQTL; *p* = 2.40 × 10^−6^) for the known AD risk gene *PICALM* (phosphatidylinositol-binding clathrin assembly protein).^41^ This variant is in perfect LD (*D’* = 1; *r*^*2*^ = 1) with a *PICALM* SNV (rs567075) previously associated with AD age at onset^42^ and is moderately correlated (*D’* = 0.93; *r*^*2*^ = 0.64) with the top *PICALM* SNV (rs3851179) from a recent large AD case-control GWAS.^7^ A strong association was also observed for rs11077216, which is immediately proximal (5 kilobases) to *RBFOX1* (RNA binding fox-1 homolog 1), a gene previously linked to amyloid PET accumulation.^14^

#### Phase 2: Refining the Candidate SNV List

In total, 274 SNVs displayed association *p* < 5 × 10^−5^ in the meta-analysis. On the assumption that this initial candidate SNV list would be overly optimistic, in part due to the inclusion of both true associations and false positives from the discovery cohorts, we integrated postmortem neuropathologic data on amyloid burden from independent samples to refine the variant list for entry to the GRS. Overall, 262 of 274 SNVs were available for analysis in ROS/MAP, including 37 SNVs displaying *p* < 0.2 and a concordant direction of effect in the neuropathology sample.

#### Phase 3: Calculating and Validating the Non-*APOE* Amyloid GRS

Using these 37 SNVs (and their β-coefficient weights from the amyloid PET meta-analysis) as the entry candidates and after LD clumping and thresholding, a GRS consisting of 6 SNVs (eTable 2) was associated with amyloid PET levels in the MCSA (*p* = 4.34 × 10^−9^, β = 5.88) and ADNI (*p* = 1.87 × 10^−8^, β = 12.1) cohorts. Compared with the standard of an AD GRS (based on variants associated with clinically diagnosed AD dementia), the non-*APOE* amyloid GRS explained a larger percentage of the phenotypic variance in both the MCSA (6.0% vs 1.0%) and ADNI (5.8% vs 0%) cohorts. In an independent sample from the MCSA (not overlapping with the amyloid PET cohort used for discovery and generation of the GRS), the non-*APOE* amyloid GRS was also associated with plasma p-tau_181_ concentrations (*p* = 0.03, β = 0.05). Among individuals who carried at least one *APOE* ɛ2 or ɛ4 allele (who were not included in the main analyses for this study), there was no association of the non-*APOE* amyloid GRS with amyloid PET levels in the MCSA and ADNI discovery samples nor with plasma p-tau_181_ concentrations in the MCSA validation sample. By contrast, a standard AD GRS (based on variants associated with clinically diagnosed AD dementia) was associated with these amyloid outcomes in each tested sample (*p* < 0.005), highlighting the specificity of the non-*APOE* amyloid GRS applying to the *APOE* ɛ3/ɛ3 setting.

#### Phase 4: Testing the Non-*APOE* Amyloid GRS for Associations With Postmortem AD Neuropathology

In *APOE* ε3/ε3 individuals from the ROS/MAP, the non-*APOE* amyloid GRS was associated with postmortem β-amyloid burden (*p* = 1.08 × 10^−4^, β = 0.67, N = 704). The non-*APOE* amyloid GRS was also associated with quantitative neurofibrillary tangle burden in ROS/MAP participants (*p* = 9.17 × 10^−4^, β = 0.10, N = 722) and with high Braak stage (≥4) in MCSA participants (*p* = 0.015, OR = 2.54 [1.19–5.39], N = 63). These findings support the ability of the non-*APOE* amyloid GRS to capture genetic risk–associated AD pathophysiology measured through in vivo biomarkers and postmortem neuropathology.

## Discussion

In this study, we conducted multicohort analyses to develop and validate a GRS for β-amyloid pathology in *APOE* ε3/ε3 older adults. This novel amyloid GRS outperformed a standard AD GRS (based on risk variants associated with risk of clinically diagnosed AD dementia) in predicting β-amyloid plaque burden across 2 cohorts with PET imaging. The non-*APOE* amyloid GRS was also associated with hallmark postmortem neuropathologic features of AD (β-amyloid plaque and neurofibrillary tangle pathology) and in an independent validation sample was associated with plasma p-tau_181_ concentrations (a robust indicator of cerebral amyloidosis). These findings support the hypothesis that there are unique genetic influences on susceptibility to β-amyloid deposition in *APOE* ε3/ε3 individuals, and that a GRS capturing these effects in aggregate may be useful for risk stratification in the general population, where most of the individuals do not carry *APOE* ε4 or ε2.

Twin studies suggest that brain β-amyloid deposition has at least moderate heritability, for which *APOE* ε4 is partly explanatory.^43^ However, it is increasingly recognized that the outsized influence of the *APOE* locus on amyloid deposition may cloud interpretation of other genetic associations (which may in fact be *APOE*-dependent despite attempts at simple covariance) while also potentially masking true effects of additional loci.^9^ A unique aspect of this study involved the focus on GRS prediction of cerebral amyloidosis (as a specific AD endophenotype) within a targeted population of *APOE* ε3/ε3 older adults, where any potential confounding influences from *APOE* ε4 or ε2 would be expected to be absent. Our data confirmed associations for previously discovered variants in *PICALM* and *RBFOX1*, findings that support the validity of this approach. Because the frequency of *APOE* ε4 varies across research cohorts analyzed in the field, our results also suggest that population-specific (e.g., ε4-positive vs ε4-negative) effects may explain a proportion of false-negative and false-positive genetic findings due to underlying heterogeneity. This implies that larger sample sizes alone may be insufficient for discovery and replication of additional genetic associations with AD and its endophenotypes, particularly if genetic heterogeneity is not addressed upfront through study design.

**Figure 3:**
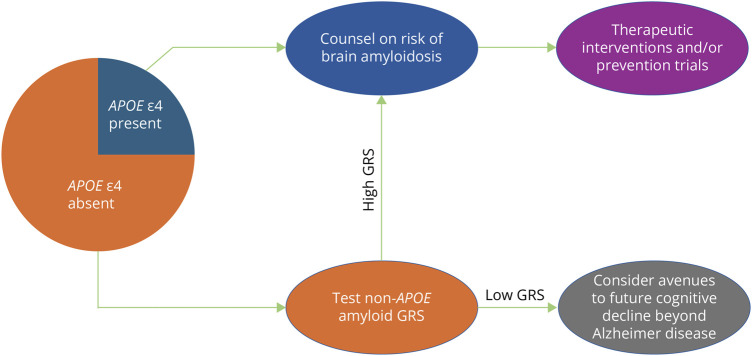
Hypothetical Model of Tiered Genetic Testing for Risk Stratification in the Population A conceptual model is presented demonstrating how genetic testing may be used in future clinical practice to guide individuals on risk of developing brain amyloidosis. In this hypothetical model, individuals carrying *APOE* ɛ4 (the strongest known individual genetic risk factor of brain amyloidosis) would be counseled on its implications and directed to potential therapeutic interventions or prevention trials. For most of the general population who do not carry *APOE* ε4, the non-*APOE* amyloid GRS would be used to stratify individuals into those who may still be at high risk of brain amyloidosis (despite being *APOE* ε4 negative) vs those for whom non-Alzheimer paths to future cognitive decline would be prioritized for consideration.

In addition to capturing loci previously found through case-control studies of clinically diagnosed AD dementia (e.g., *PICALM*), our GWAS/GRS also identified newly implicated genes with biologically plausible connections to amyloid pathways. These included *FBXO21*, which in mouse models has been demonstrated to influence ubiquitination of structures important for brain β-amyloid clearance.^44^ Overall, our results support a broader model for genetic risk stratification for amyloid deposition in the general population, whereby *APOE* ε4 and the non-*APOE* amyloid GRS exert robust but subpopulation-specific (i.e., ε4-present distinct from ε4-absent) effects while other factors including *APOE* ε2 and additional case-control AD risk alleles may more modestly influence amyloid accumulation in these states (Figure 3).

A fundamental principle underlying most GRS analyses is that individual variants may not have to exceed the stringent threshold for genome-wide significant association to meaningfully contribute toward susceptibility/resistance of a complex outcome.^45^ On the presumption that the initial results (from the MCSA/ADNI meta-analysis) would be overly optimistic for GRS calculation, we leveraged independent cohort data to prune this SNV list to include only high-likelihood true associations. It is important to emphasize that this study design is not strictly unbiased because much of the GRS generation was based on findings from the training data sets (MCSA and ADNI) and the filtering process partly selected for variants displaying a degree of association with the target outcome in the validation data sets (ROS/MAP). As a result, the estimates of variance explained by our GRS need to be interpreted with some caution. However, our analyses resulted in a GRS measure that was a strong surrogate for brain amyloid burden across multiple cohorts. It is important to note that we subsequently followed these analyses with validation studies in an independent sample with plasma p-tau_181_ data. This replication and the robustness of the GRS in predicting cerebral amyloidosis across multiple modes of measurement (PET, plasma, postmortem neuropathology) argue for further investigation in additional research samples to more deeply characterize its broader applicability in the general population.

This work has limitations. Although this study included a fairly large number of participants with amyloid markers, the sample size was still modest in comparison with most case-control GWAS/GRS studies and there remains a need for further analyses of larger data sets with amyloid pathology measures and genetic data to serve as additional validation. The cohorts analyzed here were also not perfect analogs of each other and included demographic and clinical status differences reflecting their distinct recruitment approaches. Relatedly, while efforts were made to harmonize approaches wherever possible, given the use of multiple cohorts, there remained some heterogeneity in the methods used to acquire and process genetic data used in this study. The converse of this fact of study design is that the broad consistency of our findings demonstrating the non-*APOE* GRS as a predictor of amyloid pathology represents a strength of the work overall. In addition, not every variant identified in the GWAS meta-analysis as a potential GRS element was present for analysis in the validation data sets (due to failing imputation or quality control), which may have altered the final model. To achieve true broad applicability, additional work would also be critical to assess the value of the non-*APOE* amyloid GRS in populations other than non-Hispanic Caucasian individuals, as in a recent large multiethnic GWAS of amyloid PET.^19^ Finally, a large proportion of the variance in amyloid PET levels remained unexplained by the non-*APOE* amyloid GRS in these analyses, suggesting that additional elements remain to be discovered. These factors plausibly include rare variants, sex differences, epigenetic mechanisms, environmental elements, and gene-by-environment interactions.

Through integrating multimodal data across independent cohorts, this study developed and validated a novel GRS for amyloid deposition in *APOE* ε3/ε3 individuals. The genes identified through this unique approach have the potential to offer new insights into mechanisms of amyloid accumulation across the large swath of individuals who develop AD in the absence of *APOE* ε4. Our novel GRS may also be useful for blood-based screening to identify individuals at risk of amyloidosis in future. This potential use case may have unique implications in the era of new disease-modifying therapies targeting β-amyloid plaque removal, where emerging drugs are being used clinically in individuals with early symptomatic AD and are also being tested in treatment trials in individuals at risk of future cognitive decline due to AD.^46^

## Appendix Coinvestigators

**Table:**

Coinvestigators are listed in the Supplementary Material at Neurology.org/NG

## Glossary

AD: Alzheimer disease
ADNI: Alzheimer's Disease Neuroimaging Initiative
CKD: chronic kidney disease
GRS: genetic risk score
GWAS: genome-wide association study
MAF: minor allele frequency
MAP: Memory and Aging Project
MCI: mild cognitive impairment
ROS: Rush Religious Orders Study
